# Guidelines for training in cardiovascular magnetic resonance (CMR)

**DOI:** 10.1186/s12968-018-0481-8

**Published:** 2018-08-16

**Authors:** R. J. Kim, O. P. Simonetti, M. Westwood, C. M. Kramer, A. Narang, M. G. Friedrich, A. J. Powell, J. C. Carr, J. Schulz-Menger, E. Nagel, W. S. Chan, J. Bremerich, K. G. Ordovas, R. C. Rollings, A. R. Patel, V. A. Ferrari

**Affiliations:** 1 Duke University Medical Center, Durham, USA; 2 Ohio State University, Columbus, USA; 3 Barts Heart Centre, London, UK; 4 University of Virginia Health System, Charlottesville, USA; 5 University of Chicago Medicine, Chicago, USA; 6 McGill University Health Centre, Montreal, Canada; 7 Boston Children’s Hospital, Boston, USA; 8 Northwestern University, Evanston, USA; 9 Charite Universitatsmedizin and HELIOS Clinics, Berlin, Germany; 10 Goethe University Frankfurt, Frankfurt, Germany; 11 Queen Mary Hospital, High West, Hong Kong; 12 University Hospital Basel, Basel, Switzerland; 13 University of California San Francisco, San Francisco, USA; 14 Memorial Savannah Cardiology, Savannah, USA; 15 Hospital of the University of Pennsylvania, Philadelphia, USA

**Keywords:** Cardiovascular magnetic resonance, Training, Credentialing, Certification

## Abstract

These “Guidelines for training in Cardiovascular Magnetic Resonance” were developed by the Certification Committee of the Society for Cardiovascular Magnetic Resonance (SCMR) and approved by the SCMR Board of Trustees.

## Background

Guidelines for training in cardiovascular magnetic resonance (CMR) were published by the Society for Cardiovascular Magnetic Resonance (SCMR) in 2000 [1] and updated in 2007 [2]. Since these documents were published, the clinical practice of CMR has evolved and a need arose to provide a training pathway for physicians already in practice. In response, the SCMR has developed these new training guidelines which, as before, are designed to be internationally applicable to CMR practitioners with a variety of medical backgrounds. This document was written by the Certification Committee of the SCMR which is comprised of an international group of cardiovascular radiologists, scientists, and cardiologists.

The guidelines are intended to be used by credentialing committees of healthcare facilities and agencies internationally. Additionally, this document provides the criteria to receive official verification from the SCMR that training requirements have been fulfilled. Anyone who has met the Level 1, 2 or 3 training requirements as defined in the 2007 SCMR Training Guidelines by December 31, 2018 will be eligible for official verification by the SCMR in accordance with the previous guidelines. Anyone who has not fulfilled the training requirements prior to that date will be subject to the new requirements defined in this document.

This document focuses on CMR training criteria and is not intended to describe the board certification process, which is offered by the Certification Board of Cardiovascular Magnetic Resonance (CBCMR) (www.apca.org) Official verification of Level 2 or 3 training by the SCMR is one path to board eligibility. Other criteria besides training may be required for board eligibility.

It is intended that these guidelines should be consistent with the following American Medical Association (AMA) statement: “The AMA believes that 1) individual character, training, competence, experience, and judgment should be the criteria for granting privileges in hospitals; and 2) physicians representing several specialties can and should be permitted to perform the same procedures if they meet these criteria” (Res. 26, A-77; Reaffirmed CLRPD Rep. C, A-89) [3, 4].

These guidelines are intended to be complementary to the clinical competence statement on cardiac imaging with magnetic resonance by the American College of Cardiology Foundation (ACCF) and American Heart Association (AHA) [5], the recommendations made by the American College of Radiology (ACR) [6], the revised Cardiovascular Medicine Core Cardiology Training (COCATS 4, Task Force 8) document on CMR [7], and the SPCTPD/ACC/AAP/AHA Training Guidelines for Pediatric Cardiology Fellowship Programs (Task Force 2) [8]. These guidelines are furthermore intended to be complementary to the European Diploma of Cardiovascular Radiology issued by the European Society of Cardiovascular Radiology (ESCR) as well as the CMR section of the European Association of Cardiovascular Imaging (EACVI) published curriculum [9] and European certification in CMR.

## Methods

This final version was approved by the SCMR Executive Committee and Board of Trustees.

## Recommendations for basic training in CMR

### Level 1: General training to provide a working knowledge of CMR methods and diagnostic utility

Level 1 training is intended to provide introductory knowledge in CMR. Level 1 competency represents knowledge of the indications for and capabilities of CMR, but not to the extent that is sufficient for independent practice and clinical interpretation of CMR. The topics to be covered in Level I training are listed in Table 1.

**Table 1:** Topics to be covered in Level 1 training

• CMR safety: magnetic fields, and contraindications	
• CMR contrast agents: mechanisms, safety, and applications	
• Blood flow imaging and quantitative analysis of velocity-encoded images	
• CMR of cardiovascular structure and anatomy	
• CMR of right and left ventricular function	
• CMR of myocardial infarction, scarring, and viability assessment	
• CMR of myocardial fibrosis and edema	
• CMR stress testing	
• CMR of vascular pathology, including aortic disease	
• CMR of valvular heart disease	
• CMR of pediatric and adult congenital heart disease	
• CMR in cardiomyopathies and heart failure	
• CMR in arrhythmia	
• CMR of pericardial disease	
• CMR for cardiac masses and tumors	

Level 1 training requirements can be met through either of two tracks. Track A is tailored for fellows or residents in training, and Track B is designed for physicians who have completed medical training. The mentored cases and time spent in training in Track A can also be applied towards Level 2 training requirements.

#### Track A (formal fellowship or residency training)

It is recommended that all training programs (adult and pediatric cardiovascular medicine, radiology, and nuclear medicine) incorporate basic training in CMR to familiarize the trainee with the indications, methods, and applications of CMR. Level 1 CMR training can be completed by meeting the following requirements, typically during the course of residency or fellowship training:

- A minimum of 4 weeks of cumulative (need not be consecutive) exposure to the methods and applications of CMR.
- Training must be under the aegis of a Level 2 or preferably Level 3 qualified mentor.
- Training must provide exposure to the topics listed in Table 1.
- Exposure to a minimum of 50 comprehensive mentored cases.

Official verification of SCMR Level 1 training may be sought by providing a letter from the trainee’s laboratory director verifying that the above requirements have been met.

#### Track B (course-based training)

This track is designed primarily for physicians who have completed medical training. Level 1 CMR training can be completed by meeting the following requirements, typically attained during a dedicated CMR training course:

- The course must include a minimum of 2 full days of dedicated, live instruction in the indications, methods, and applications of CMR.
- The course director must be Level 2 or preferably Level 3 qualified.
- The course must cover the topics listed in Table 1. These topics may be presented as either didactic lecture or as case studies.

Official verification of SCMR Level 1 training may be sought by providing the course syllabus and a letter from the course director verifying that the above requirements have been met.

## Criteria for the practice of CMR

### Level 2: Specialized training designed to provide the skills necessary to independently interpret CMR studies

For a physician to perform and interpret CMR independently, the following criteria (divided into general and specific categories) are required:

#### General criteria

- Hold a valid unrestricted medical license or registration.
- Board certification or equivalent specialist medical qualification in adult or pediatric cardiovascular medicine, adult or pediatric radiology, or nuclear medicine.

OR

- Completion of an accredited training program and board eligibility or equivalent in adult or pediatric cardiovascular medicine, adult or pediatric radiology, or nuclear medicine.

#### Specific criteria

Initial training:

- At least 12 weeks of dedicated CMR training under the supervision of a Level 2 or Level 3 (preferred) qualified mentor including a minimum of 6 weeks full-time equivalent training in a CMR laboratory and up to 6 weeks of training outside of a CMR laboratory. Both in-lab and out-of-lab training time is defined as a minimum of 35 h/week and may be spread over more than 12 weeks as long as the total time requirements are met. Training outside of a CMR laboratory may consist of coursework or case studies provided on-line or via CD/DVD, time at major medical meetings that have significant content devoted to CMR, or remote mentoring. The 12 weeks of dedicated CMR training need not be continuous or at a single institution but must be completed within 4 years.
- At least 50 h of CMR related coursework within the training program, or external CME certified coursework within the timeframe of the training program.
- Supervised interpretation of at least 150 CMR studies representing the range of CMR applications and pathologies observed in practice. CMR studies should include a mix of the cardiac and vascular indications listed in Table 1. For at least 50 studies, the trainee must be present during the scan as the primary operator or alongside the technologist, perform the image analysis, and make the initial interpretation. Supervised interpretation means that detailed feedback of the interpretation is provided by a SCMR Level 2 or Level 3 or international equivalent qualified mentor.

Maintenance of skills:

- CME in CMR for at least 20 h every 2 years (subject to audit).
- Primary interpretation of at least 100 cases every 2 years (subject to audit).

## Criteria for advanced training in CMR

### Level 3: Advanced training for those who ultimately wish to be responsible for the operation of a CMR laboratory and to participate in CMR teaching and research

Initial training:

In addition to Level 2 general criteria and training:

- A total of at least 12 months training in CMR under the aegis of a Level 3 qualified mentor to be completed within 3 years.
- Supervised interpretation of a total of at least 300 CMR studies representing the range of CMR applications and pathologies observed in practice. For at least 100 studies, the trainee must be present during the scan as the primary operator or alongside the technologist, perform the image analysis, and make the initial interpretation.
- Active participation in the local quality assurance or improvement program for the acquisition, post-processing, and reporting of CMR studies.
- Active participation in basic or clinical CMR research or teaching during the training period.

Maintenance of skills:

- CME in CMR for at least 40 h every 2 years (subject to audit).
- Be the primary interpreter of at least 200 cases every 2 years (subject to audit).

## Proof of training for Level 2 and Level 3

The CMR training center and the trainee should maintain a logbook or other specific records to document the duration of the trainee’s in-lab and out-of-lab training, the number of case reviews, and didactic hours.

Official SCMR verification of completion of Level 2 or Level 3 training may be sought by providing a letter or certificate from the trainee’s laboratory director or from the director of their fellowship training program verifying that the relevant training level has been achieved.

## Legacy criteria for Level 2 and Level 3

An individual may be exempted from the above training requirements if the following criteria were met prior to July 1, 2003.

- Board certification in cardiovascular medicine, radiology, or nuclear medicine.
- Two years or more of substantial activities in CMR including at least two of the following:
  - ○ Documented involvement in the performance and interpretation of at least 200 CMR studies.
  - ○ CMR research activities verified by publication record.
  - ○ Acknowledged clinical teacher of CMR with at least two or more trainees.
- Active participation in CME and local quality assurance programs as required for Level 2 or Level 3 requirements above.

SCMR verification that the equivalent of Level 2 or Level 3 training was achieved via this legacy pathway may be sought by submitting proof that the above criteria were met prior to July 1st 2003. Individuals who qualify for this legacy pathway must document their maintenance of skills as defined in the Level 2 and Level 3 requirements above.

## Conclusion

Not applicable.

## Abbreviations

AAP: American Academy of Pediatrics
ACC: American College of Cardiology
ACCF: American College of Cardiology Foundation
ACR: American College of Radiology
AHA: American Heart Association
AMA: American Medical Association
CBCMR: Certification Board of Cardiovascular Magnetic Resonance
CMR: Cardiovascular Magnetic Resonance
COCATS: Core Cardiovascular Training Statement
EACVI: European Association of Cardiovascular Imaging
ESCR: European Society of Cardiovascular Radiology
SCMR: Society for Cardiovascular Magnetic Resonance
SPCTPD: The Society of Pediatric Cardiology Training Program Directors
